# A trial protocol for the effectiveness of digital interventions for preventing depression in adolescents: The Future Proofing Study

**DOI:** 10.1186/s13063-019-3901-7

**Published:** 2020-01-02

**Authors:** Aliza Werner-Seidler, Kit Huckvale, Mark E. Larsen, Alison L. Calear, Kate Maston, Lara Johnston, Michelle Torok, Bridianne O’Dea, Philip J. Batterham, Susanne Schweizer, S. Rachel Skinner, Katharine Steinbeck, Julie Ratcliffe, Ju-Lee Oei, George Patton, Iana Wong, Joanne Beames, Quincy J. J. Wong, Raghu Lingam, Katherine Boydell, Allison M. Salmon, Nicole Cockayne, Andrew Mackinnon, Helen Christensen

**Affiliations:** 1 Black Dog Institute, University of New South Wales, Sydney, NSW Australia; 2 Centre for Mental Health Research, The Australian National University, Canberra, ACT Australia; 3 School of Psychology, University of New South Wales, Sydney, NSW Australia; 4 Institute of Cognitive Neuroscience, University College London, London, UK; 5 Discipline of Child and Adolescent Health, Faculty of Medicine and Health, The University of Sydney, Sydney, NSW Australia; 6 College of Nursing and Health Sciences, Flinders University, Adelaide, SA Australia; 7 School of Women’s and Children’s Health, University of New South Wales, Sydney, NSW Australia; 8 Murdoch Children’s Research Institute and University of Melbourne, Melbourne, VIC Australia; 9 School of Social Sciences and Psychology, Western Sydney University, Sydney, NSW Australia; 10 Population Child Health Clinical Research Group, School of Women’s and Children’s Health, University of New South Wales, Sydney, NSW Australia

**Keywords:** Depression, Prevention, Adolescents, School, Mental health, eHealth, mHealth, Digital health, Smartphone application, Cognitive behaviour therapy

## Abstract

**Background:**

Depression frequently first emerges during adolescence, and one in five young people will experience an episode of depression by the age of 18 years. Despite advances in treatment, there has been limited progress in addressing the burden at a population level. Accordingly, there has been growing interest in prevention approaches as an additional pathway to address depression. Depression can be prevented using evidence-based psychological programmes. However, barriers to implementing and accessing these programmes remain, typically reflecting a requirement for delivery by clinical experts and high associated delivery costs. Digital technologies, specifically smartphones, are now considered a key strategy to overcome the barriers inhibiting access to mental health programmes. The Future Proofing Study is a large-scale school-based trial investigating whether cognitive behaviour therapies (CBT) delivered by smartphone application can prevent depression.

**Methods:**

A randomised controlled trial targeting up to 10,000 Year 8 Australian secondary school students will be conducted. In Stage I, schools will be randomised at the cluster level either to receive the CBT intervention app (SPARX) or to a non-active control group comparator. The primary outcome will be symptoms of depression, and secondary outcomes include psychological distress, anxiety and insomnia. At the 12-month follow-up, participants in the intervention arm with elevated depressive symptoms will participate in an individual-level randomised controlled trial (Stage II) and be randomised to receive a second CBT app which targets sleep difficulties (Sleep Ninja) or a control condition. Assessments will occur post intervention (both trial stages) and at 6, 12, 24, 36, 48 and 60 months post baseline. Primary analyses will use an intention-to-treat approach and compare changes in symptoms from baseline to follow-up relative to the control group using mixed-effect models.

**Discussion:**

This is the first trial testing the effectiveness of smartphone apps delivered to school students to prevent depression at scale. Results from this trial will provide much-needed insight into the feasibility of this approach. They stand to inform policy and commission decisions concerning if and how such programmes should be deployed in school-based settings in Australia and beyond.

**Trial registration:**

Australian and New Zealand Clinical Trial Registry, ACTRN12619000855123. Registered on 31 May 2019.

Clinical Trial Notification Scheme (CTN), CT-2019-CTN-02110-1-v1. Registered on 30 June 2019.

## Background

According to the World Health Organization, depression is the leading cause of disability worldwide [1]. For many individuals, depression first emerges during adolescence [2], between age 10 and 24 years [3]. Approximately one in five young people will experience a depressive episode by the age of 18 years [4], with earlier onset being associated with a range of adverse consequences including poor academic performance, low levels of school attendance, social dysfunction, substance abuse, anxiety, suicidality, poor sleep, diabetes and metabolic syndrome [5–7]. As adolescents develop, the foundations are laid for successful transition into the varied roles of adulthood including the time in life when employment productivity peaks. Experiencing depression as a young person can negatively impact psychological, social, emotional, educational and vocational pathways into adulthood, with profound implications for the individual, their families and their communities, as well as the economy [5, 8]. The global cost of mental illness is nearly US$2.5 trillion, two-thirds of which is incurred via indirect costs such as unemployment. This cost is expected to increase to US$6000,000,000,000 by 2030 [9, 10]. By way of benchmarking, total global health expenditure in 2009 was US$5.1 trillion, of which less than 2% is spent on mental health [11]. The immense cost of mental illness, together with the social and emotional consequences associated with earlier onset, highlights the crucial importance of prevention or intervention early in the course of illness, and, consequently, at an early age.

There has been little progress in recent years addressing this challenge. Estimates suggest that only 13% of the disease burden due to depression can be alleviated through current treatments and service models [12]. Even with optimal clinician competence, improved access and treatment adherence, only 36% of the burden is avoidable, leaving substantial unaddressed morbidity and disability [12]. Currently, fewer than one-third of young people seek help and gain access to first-line interventions for reasons that include accessibility, stigma and affordability [13]. Of those who seek professional help, between 30 and 50% of young people do not respond to treatment [14], and among those who do, over 45% will experience a subsequent depressive episode [15]. This has led to growing recognition that treatment alone will not successfully reduce the burden of depression at a population level [16].

Prevention represents a separate pathway through which the burden of depression may be addressed. There has been bourgeoning interest in the use of preventive approaches, with several high-quality but small-scale prevention studies demonstrating that the prevention of depression is possible in school-aged young people. Several recent meta-analyses have examined the effect of psychological prevention programmes specifically on adolescents and found consistent but small effect sizes in favour of delivering CBT for depression, at least in the short term [17–19]. Of the prevention programmes that are available, those based on cognitive behaviour therapy (CBT) have been the most frequently evaluated interventions, with evidence to support these approaches [20]. Other approaches may prove effective but have not yet been subject to rigorous empirical evaluation. CBT involves challenging unhelpful thoughts or beliefs, by gathering evidence which contradict these thoughts or beliefs from real-world situations. This leads to changes in emotion and behaviour. CBT is structured, brief and time-limited, and aims to help individuals develop skills and strategies to manage their own mood and depression. Prevention approaches can be delivered either universally to all individuals within an identified population regardless of risk (universal prevention), or targeted to those with risk factors such as low socio-economic background (selective prevention) or to those who have sub-threshold disorder symptoms (indicated prevention). While there is evidence for all three prevention approaches [18, 19, 21], there have typically been larger effects reported for targeted approaches, at least in part due to limitations in the statistical power of universal prevention studies [22].

Despite consistent statistically and clinically meaningful effects in existing prevention studies, these studies have been limited by several factors. First, trials have generally involved small sample sizes, and the consequent limited statistical power means that potential effects go undetected and/or are measured imprecisely. Specifically, the median sample size identified in a recent meta-analysis of 47 studies examining the prevention of depression was 208, with an average effect size of *g* = 0.23 [19]. Samples of this size are insufficient because preventive programmes, particularly universally delivered programmes, necessarily expose a large proportion of individuals not at risk to the intervention, and yield small to modest effects because of the low base rates of disorder in general population samples [21]. Second, studies have rarely involved follow-up beyond 12 months, which limits what is known about long-term preventive effects. Third, studies have relied solely on self-report or clinician-reported symptoms. While subjective reports reflect the gold-standard approach and are useful clinically, there is much to be gained by the inclusion of additional objectively quantifiable data sources (e.g., health care utilisation data, smartphone-collected sensor data). Fourth, most prevention approaches involve the delivery of a single intervention or programme. There is rarely an option to identify who is most likely to respond to different interventions, or for a tiered approach whereby individuals for whom one approach has not been effective may try a different approach afterwards. This is potentially a missed opportunity given that not everyone will respond to a single, or the first-line, approach. Finally, CBT has, until recently, been delivered to young people generally over 6–12 sessions using face-to-face approaches which are challenging to scale due to the expertise and cost required to deliver such programmes. Digital approaches may be easier to scale up; however, in a review of 146 randomised controlled prevention trials, only two involved digitally delivered interventions [18]. In light of these limitations, there is scope to improve on and extend the empirical base to understand the effects of depression prevention programmes.

### The current study

The Future Proofing Study has been designed to overcome these challenges by conducting a large school-based randomised controlled trial (RCT) testing whether depression can be prevented using a CBT programme delivered by smartphone application. Research evidence to date suggests that digital CBT programmes for depression delivered in schools can be effective in reducing and preventing depression [23]. Partnering in the delivery of prevention programmes is a natural fit for schools, which are increasingly seen as settings in which to foster emotional skill development alongside traditional academic learning. Mental ill health is associated with poorer academic performance [24], and depressed young people miss an average of 22 days of school per year [6], making mental well-being in line with the goals of educators and school administrators. Importantly, schools provide access to large student samples which are more representative of the general population than other settings, such as mental health services or medical practices. In addition, the potential to deliver a prevention programme to all students universally may help reduce the stigma often associated with the delivery of mental health interventions only to those with symptoms [13].

This trial will involve up to 10,000 Australian school students who will be followed up for 5 years. A sample of this size provides appropriate statistical power to definitively and precisely evaluate effectiveness, as well as providing the opportunity to conduct robust moderation and mediation analyses. All study protocols will be delivered to participants digitally. The intervention/s will be delivered by smartphone application (apps) and study surveys can be completed on any Internet-enabled device (smartphone, tablet, laptop). This delivery method overcomes the aforementioned challenges associated with delivery by trained health professionals or teachers. It is simply too costly to train enough of the workforce to deliver CBT prevention programmes either individually or in small groups to make an impact at a population level. Delivering interventions digitally is more cost-effective than face-to-face therapies [25]. Moreover, programmes using a smartphone app for delivery do not compromise effectiveness [26]. Testing smartphone apps represents a major innovation of this project, with most evaluation of digital programmes focusing on Internet-based approaches [27]. Digital technologies, and specifically smartphones, are now considered a key strategy to overcome the barriers inhibiting access to mental health programmes. This mode of delivery is appropriate given that young people show a preference for digital technologies when given the choice, due to the anonymity, convenience and privacy [12, 28].

This SPIRIT-compliant protocol describes the methodology for a multi-stage, two-arm, parallel group RCT (Additional file 1). At Stage I, a cluster RCT will be undertaken to evaluate the effectiveness of SPARX, which is an app-based CBT gaming intervention [29]. This intervention has been tested in the Australian adolescent population and was found to be effective for the prevention of depression [23]. In the current trial, SPARX will be delivered to Year 8 students (who are typically aged 12–14 years). The control group will consist of Year 8 students of an equivalent demographic who will not receive any intervention. Participants across both arms will be followed longitudinally over 5 years. The choice to use a non-active (treatment-as-usual) comparator was made to avoid placing unnecessary burden on the participants and schools, which would have been required to provide an attention-matched placebo intervention—an intervention that would have required an equivalent level of effort to that of the active condition—that would most likely provide no benefit. Other candidate programmes that may have benefited other outcomes such as lifestyle or nutrition programmes could not serve as a fair control condition because of their likely impact on mental health. At Stage II (12 months following baseline), an individual-level RCT will be conducted within the intervention arm. Individuals who show elevated symptoms of depression at 12 months will be randomised to an intervention condition that receives a second CBT app which targets sleep difficulties (Sleep Ninja) or a control condition. Sleep Ninja focuses on sleep disturbances and delivers cognitive behaviour therapy for insomnia (CBT-I). An intervention targeting sleep difficulties was selected because sleep disturbance is both a risk factor and a symptom of depression, with efficacy supporting its use in the prevention and treatment of depression in both adults and adolescents [28, 30–32].

### Aims

This trial will investigate the impact of two mental health apps on adolescent depression. The first objective of this trial is to determine whether delivering a CBT programme via smartphone application can prevent high school students in Year 8 from developing depressive symptoms compared to students in the control arm. This study will assess the effect of the programme on a range of secondary outcomes, which include mental health symptoms (distress, anxiety, insomnia) at post intervention, 6, 12, 24, 36, 48 and 60 months. The effect of a second intervention on depression in those who show elevated depressive symptoms 1 year after using the first SPARX app will also be evaluated. Individual differences associated with response to the interventions will also be investigated, including demographics, history of mental illness, personality factors, social support, social media use and gender identification, among a range of other variables (see Table 1). In the control group, these factors will be examined as possible predictors for the development of depressive symptoms and other mental health problems.

**Table 1:** Summary of primary, secondary and tertiary outcome measures, potential mediating and risk factors, and data collection time points

Outcome measure		Baseline	Post (Stage I)	6 months	12months	Post (Stage II)^a^	24 months	36 months	48 months	60 months
Primary outcome measure										
Depression	Patient Health Questionnaire: Adolescent Version (PHQ-A)	✓	✓	✓	✓	✓	✓	✓	✓	✓
Secondary outcome measures										
Psychological distress	Distress Questionnaire 5 (DQ5)	✓	✓	–	✓	–	✓	✓	✓	✓
Anxiety	Spence Children’s Anxiety Scale (SCAS) including Generalized Anxiety and Social Phobia subscales	✓	✓	–	✓	✓	✓	✓	✓	✓
Insomnia	Insomnia Severity Index (ISI)	✓	✓	–	✓	✓	✓	✓	✓	✓
Tertiary outcome measures, potential mediating variable risk factors										
Suicidal ideation	Suicidal Ideation Attributes Scale (SIDAS)	✓	✓	–	✓	–	✓	✓	✓	✓
Suicide behaviour	Youth Risk Behaviour Survey (YRBS)	✓	✓	–	✓	–	✓	✓	✓	✓
Externalising behaviours	Strength and Difficulties Questionnaire (SDQ)	✓	✓	–	✓	–	✓	✓	✓	✓
Eating disorders	Screen for Disordered Eating (SDE)	✓	✓	–	✓	–	✓	✓	✓	✓
Self-harm	Single question from Self-Harm Questionnaire	✓	✓	–	✓	–	✓	✓	✓	✓
Alcohol use	Questions from previous National Drug and Alcohol Research Centre, UNSW studies	✓	✓	–	✓	–	✓	✓	✓	✓
Other substance use	Questions from Australian Institute of Health and Welfare 2007 National Drug Strategy Household Survey	✓	✓	–	✓	–	✓	✓	✓	✓
Psychosis	Adolescent Psychotic-like Symptom Screener (APSS)	✓	✓	–	✓	–	✓	✓	✓	✓
Sleep quality	Pittsburgh Sleep Quality Index (PSQI)	✓	✓	–	✓	✓	✓	✓	✓	✓
Quality of life	Child Health Utility 9D (CHU-9D)	✓	✓	–	✓	–	✓	✓	✓	✓
Well-being	Short-form Warwick–Edinburgh Mental Well-Being Scale (SWEMWBS)	✓	✓	–	✓	–	✓	✓	✓	✓
Demographics	Age, ethnicity, location, language, perceived socio-economic status	✓	–	–	–	–	–	–	–	–
Height and weight	Height (cm), weight (kg)	✓	–	–	✓	–	✓	✓	✓	✓
Personality	Big Five Inventory-10 (BFI-10)	✓	–	–	–	–	–	–	–	–
School connectedness	Items from OECD Programme for International Student Assessment (PISA)	✓	✓	–	✓	–	✓	✓	✓	–
Social support	Schuster Social Support Scale (SSSS)	✓	✓	–	✓	–	✓	✓	✓	✓
Social media use	Maladaptive Facebook Use Survey (MFUS), adapted to apply to social media use more broadly	✓	✓	–	✓	–	✓	✓	✓	✓
Gender identification	Sex at birth and current gender identification	✓	–	–	✓	–	✓	✓	✓	✓
Sexual identification and preferences	Single item for each	✓	–	–	✓	–	✓	✓	✓	✓
Sexual behaviour	History of sexual behaviour	–	–	–	–	–	✓		✓	
Romantic relationships	History of romantic relationships	✓			✓		✓	✓	✓	✓
Trauma	Adapted Behavioural Risk Factor Surveillance System—Adverse Childhood Experience (BRFSS-ACE)	✓ no abuse items	–	–	–	–	–	–	✓	–
History of diagnosed mental health condition	Diagnosed mental health conditions	✓	✓	–	✓	–	–	–	✓	✓
Hospitalisation	History of hospitalisation	✓	✓	–	✓	–	–	–	✓	✓
History of diagnosed disability	Diagnosed disability	✓	–	–	–	–	–	–	✓	–
Pubertal development	Menarche, voice-breaking and Tanner stages	✓	–	–	✓	–	✓	✓	✓	✓
Bullying	From previous school trials	✓	✓	–	✓	–	✓	✓	✓	✓
App feedback/acceptability										
App feedback	Future Proofing App Feedback Survey	–	✓	–	–	–	–	–	–	–
App feedback	Post-intervention SPARX-FP Feedback Survey	–	✓	–	–	–	–	–	–	–

*UNSW* University of New South Wales

^a^Participants selected to take part in Stage II of the trial only

The second objective of the trial is to identify the predictors of mental health outcomes from digital behavioural and cognitive data collected using participants’ smartphones (see Table 2 for details). Sensor data will be collected from smartphones to provide information about activity levels (accelerometery), movement through space (GPS) and voice patterns to generate a ‘digital phenotype’ for each participant [33–35]. Digital phenotyping can be understood as using sensor data collected by personal devices to quantify the individual-level human phenotype. One of the functions of profiling individuals using digital phenotypes in this way is to make predictions about changes in mental health. The sensors in smartphones provide a non-intrusive, minimally burdensome and scalable method to investigate the effect of activity and behaviour on mental health. Evidence of the validity of this approach is beginning to emerge, with location variability now showing an association with depression outcomes in small studies of adults [36, 37]. However, the use of digital phenotyping approaches in young people at scale has not yet been undertaken. For participants in the intervention group, this information will be combined with intervention usage data and other study measures to explore predictors of response and non-response to the intervention apps. In the control group, this information will be used to prospectively examine predictors of psychopathology.

**Table 2:** Future Proofing app tasks, data and collection time points

Smartphone-collected data	Approximate time	Baseline	Week 1	Week 2	Week 3	Week 4	Week 5	Week 6	6 months	12 months	24 months	36 months	48 months	60 months
Self-rated mood visual analogue scale (VAS)	1 min	•	•	•	•	•	•	•	•	•	•	•	•	•
Affective card sorting task (CST) cognitive game	3 min	•	•	•	•	•	•	•	•	•	•	•	•	•
Affective backward span task (BST) cognitive game	3 min	•	•	•	•	•	•	•	•	•	•	•	•	•
Speed of typing task	2 min	•	•	•	•	•	•	•	•	•	•	•	•	•
Voice sample task	2 min	•		•		•		•	•	•	•	•	•	•
Accelerometery (movement) device sensor data	3 months of continuous data collection													
Gyroscope (movement) device sensor data	3 months of continuous data collection													
GPS (location) device sensor data	3 months of continuous data collection													

The third objective of this trial is to evaluate the implementation of the proposed CBT-based depression prevention programme in the school environment. The data that will be collected to meet this objective will combine intervention usage with qualitative and quantitative methods. A protocol for the process evaluation of the programme implementation will be detailed elsewhere.

Three different forms of data will be collected from this trial to address each of the objectives: self-report questionnaires (first objective), smartphone-recorded sensor data and ecological momentary assessment data (second objective), and usage and implementation data (third objective). This study will also utilise health and education datasets as a fourth form of data that will be linked to the main study datasets 1 year after the trial commences, and then periodically until the trial is complete. Linked data will include academic outcomes, health service utilisation and infant development, alongside births and deaths records. Linkage will allow for a more complete picture of the education outcomes and the health of the sample and a cost-effectiveness analysis.

The first form of data (self-report questionnaire data) will be reported in the primary outcome paper and limited to primary and secondary outcomes. All other data types will be reported and published separately.

### Hypotheses

The primary hypothesis is that participants allocated to the intervention arm will show a smaller increase in depressive symptoms relative to those in the control group at 12 months, measured using the self-report Patient Health Questionnaire—Adolescent Version (PHQ-A) [38]. Secondary hypotheses are that depressive symptoms will be lower (e.g., show a smaller increase) in the intervention arm relative to the control arm at other assessment points (post intervention, 6, 24, 36, 48 and 60 months). Using the pre-defined clinical cut-off points on the PHQ-A will also allow a comparison between likely cases of depression across the two arms. It is hypothesised that the percentage of depression cases will be lower in the intervention arm relative to the control arm at post intervention, 6, 12, 24, 36, 48 and 60 months. Secondary mental health outcomes are psychological distress, anxiety and insomnia, and are expected to be lower in the intervention arm relative to the control, at post intervention, 12, 24, 36, 48 and 60 months. Additional outcomes including suicidality, substance use and sleep quality will be reported separately.

For participants in the intervention condition who show clinically significant depressive symptoms at 12 months (PHQ-A score > 10) and who enter Stage II of the trial, it is hypothesised that those who receive the Sleep Ninja intervention will report greater decreases in symptoms of depression (primary outcome), anxiety and insomnia (secondary outcomes) from pre to post intervention (Stage II) and from 12 to 24 months (after they receive the Sleep Ninja intervention), relative to the Stage II control arm.

## Methods

### Trial design

This study is a randomised controlled, single-blind trial with two intervention stages, each consisting of two parallel arms (intervention and inactive control) with 1:1 allocation at each stage. Cluster randomisation will occur at the school level for the first intervention stage, stratified by school size, school location, school student gender and socio-economic status (full details in Randomisation section).

The second intervention stage will occur directly after the 12-month follow-up and involve individual-level randomisation. Stage I of the trial will have eight measurement occasions: baseline (pre-intervention); Stage I post intervention (immediately after completing the Stage I intervention, 6 weeks after baseline); and 6-month, 12-month (primary endpoint), 24-month, 36-month, 48-month and 60-month follow-up. The follow-up period is the time since baseline. For the subset of participants involved in Stage II of the trial, an additional measurement occasion will take place 6 weeks after the Stage II intervention (Stage II post intervention). The trial will take place in three recruitment waves. It was planned that Wave 1 will commence in Term 3, 2019 (July 2019), Wave 2 will commence in Term 2, 2020 (April 2020) and Wave 3 will commence in Term 3, 2020 (July 2020). The Stage I intervention phase (SPARX) will last 6 weeks and have a follow-up period of 5 years. The Stage II intervention phase (Sleep Ninja) will last 6 weeks, occurs 12 months after Stage I (SPARX) and will have a follow-up period of up to 4 years.

Data from each assessment point will be used to determine safety and whether any modifications to the trial protocol are required (see Analysis section for details). A concurrent implementation process evaluation will be conducted, and study processes may be modified to better address the needs of the school context. Any change will be reflected in the study protocol and registration (ACTRN12619000855123).

### Setting

This trial will be conducted in approximately 200 schools located predominantly in New South Wales, Australia. The school system in Australia is divided into three main school types: government schools, independent schools and Catholic schools. Schools from each of these groups will be recruited into the study. Schools across metropolitan, regional and rural locations will be invited to participate in order to generate a demographically representative sample. Similarly, schools with differing indices of community socio-educational advantage (ICSEA) will be targeted. If recruitment targets are not met, schools in Australian states outside New South Wales will be invited to participate. Recruitment is broadly aligned to the academic calendar, with delivery occurring during Term 2 (April) and Term 3 (July) such that assessment sessions take place during the school calendar year and risk issues can be addressed without delay. The first trial recruitment wave will be conducted primarily in independent schools in metropolitan Sydney and the Central Coast for reasons of convenience. The second and third waves will include both metropolitan and non-metropolitan sites, and include government, Catholic and independent schools. Trial management will take place at the Black Dog Institute, a translational research institute located in Sydney, Australia that is affiliated with the University of New South Wales (UNSW).

### Participants

As this is a universal prevention study, there are no exclusion criteria. All adolescents enrolled in Year 8 at each participating school are eligible to participate in the trial if they have a smartphone with iOS or Android operating system and an active mobile phone number. The usual age range of students in Year 8 is 12–14 years, although Year 8 students outside this age range will not be excluded.

### Research team roles and responsibilities

Several committees have been established to support the overall trial governance, each with their own charter and roles. The most senior committee is the Internal Steering Committee comprising senior investigators and staff located at the Black Dog Institute (HC, AW-S, KH, KM, AMS, NC). This group meets fortnightly and is responsible for guidance and decision-making about the overall trial design, intellectual contribution to the scientific quality and strategy, oversight of trial progress and compliance with good clinical practice. The day-to-day trial leadership team meets weekly (AW-S, KH, KM) and leads the operational aspects of the trial, including management of teams responsible for recruitment and consent (AW-S, KM, LJ) and data management data privacy and security (KH). A larger Investigator Committee comprises 20 investigators and collaborators on the project who meet annually to provide strategic guidance and input.

### Youth and public involvement

Young people, parents and consumers have been consulted in the preparation phase for the current study. Recruitment processes and study materials were reviewed by the Lived Experience Advisory Panel at the Black Dog Institute, and a local school’s Parents & Citizens (P&C) Association, with changes made based on suggestions provided. Young people, both through the Black Dog Institute network and a local mental health service youth reference group, have been involved in reviewing all of the questionnaires and informed the wording of questionnaires and explanation of concepts using ‘explainers’ to aid clarity. The two interventions have been subject to youth feedback in previous trials and iterations of the programmes. For example, young people who have previously used the SPARX intervention provided acceptability feedback in a previous trial [23] while Sleep Ninja was developed in collaboration with young people, and a sample of 50 young people provided feedback about the intervention in acceptability surveys and interviews [32].

### Recruitment

**Fig. 1:**
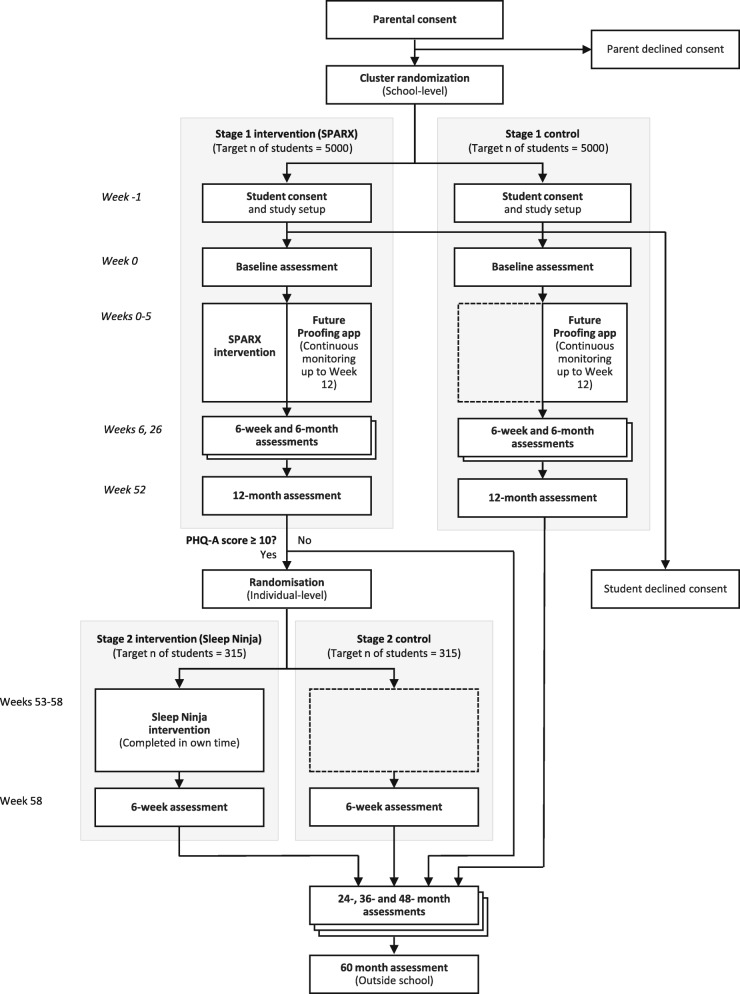
Study flow

A flowchart outlining recruitment into the trial, randomisation at trial Stages I and II, the study timeline and participation is outlined in Fig. 1. Approximately 200 schools will be recruited to participate in the trial via several recruitment pathways. The characteristics of the sample will be compared to school census data to determine representativeness. Schools will be recruited in three waves over 2 years: one wave for commencement of the trial in Term 3, 2019 (12 schools), and then another two waves in Terms 2 and 3, 2020 respectively (approximately 188 schools).

School principals will be contacted in the first instance. They will be given brief information sheets containing trial details. Schools will have the opportunity to hear more information over the telephone or via video conference. Those who wish to take part will be instructed to sign and return a letter of support. Upon receipt of the letter, schools will be accepted into the trial. In some instances, schools may approach the Black Dog Institute by submitting an expression of interest via a link on the study website (www.futureproofing.org.au) or by directly contacting staff at the Black Dog Institute.

After schools have consented, parents and students will be informed about the trial by their school via email or newsletter, depending on school preferences. A study invitation will be circulated to parents. This will include a link to an electronic information sheet and consent form. Here, parents and students can read information about the study, download a PDF copy of the study information sheet and provide electronic consent. Fourteen days following consent form distribution, schools with capacity will make follow-up telephone calls to parents who have not submitted a response. The option to provide verbal consent will be given to these parents. Students whose parents have consented to their participation will be given the opportunity to provide consent electronically at the start of the first study session. On the consent form, participants indicate whether they consent to different components of the study, including the sensor data collection and the linkage component, and whether they provide permission for their data to be included in a data repository for use by other researchers. Consent forms are available from the study website (www.futureproofing.org.au). No biological specimens will be collected. Students who do not provide active consent cannot participate in the trial. If a participant withdraws from the study, they are asked to indicate whether they give permission for their already provided data to be used by the study team. All recruitment strategies have ethical approval.

The Black Dog Institute has existing relationships with 100 schools in New South Wales, both through previous research studies and via delivery of community mental health literacy programmes to high school students. These relationships will be leveraged to support school recruitment. To maximise school consent rates, potential schools will be approached at least one school term in advance of study implementation. This means that schools can schedule study activities well in advance. Schools who participate in Wave 1 of the study will also be eligible to participate in Waves 2 or 3 (as these occur in the subsequent year and therefore involve a different cohort). As a universal study, schools will be encouraged to organise data collection assessment sessions as whole-of-classroom activities. To maximise parental consent rates, an information video specifically designed for parents will be made available, and parents have the option to directly contact the research team for further study information.

### Interventions

This trial will involve two interventions: a universal prevention intervention (Stage I; SPARX) and an indicated prevention intervention (Stage II; Sleep Ninja) for participants showing elevated depressive symptoms 12 months after receiving the universal intervention. Participants can begin or continue with any existing treatments during the study, including antidepressants and psychotherapy, and may receive additional support from health professionals if required.

#### SPARX

SPARX is a gaming intervention that was developed as a treatment programme for mild–moderate depression [29]. In its original form as a computerised game, research has shown SPARX to be effective in the treatment of adolescent depression, and equally effective as treatment as usual, which primarily involved face-to-face psychotherapy with a mental health professional [29]. This trial will use SPARX-Future Proofing (SPARX-FP; referred to as SPARX in all other sections of the document), which is an adaptation of SPARX, to make it suitable for the *prevention* of depression, for delivery via mobile phone application. The content is the same, but instead of a focus on existing symptoms and depression, terminology has been updated to focus on times when the participants have felt ‘down or stressed’ and included skills and strategies reframed to focus on dealing with problems as they arise. The prevention version of SPARX has been trialled in an Australian sample of 540 final-year secondary school students. This trial showed that, relative to a control group, those who used the programme showed lower symptoms of depression in the lead up to final school examinations [23]. The move to a mobile phone application from a web-based programme was informed by this previous study finding technological barriers to and participant preferences for delivery via smartphone.

SPARX consists of seven modules (levels) which cover: finding hope, being active, dealing with strong emotions, problem-solving, recognising unhelpful thoughts, challenging unhelpful thoughts and bringing it all together. Each module takes approximately 20 min to complete. Each module is designed to be completed on separate days, and users are encouraged to do one or two modules per week. Participants will have 6 weeks to complete the SPARX programme on their personal smartphones, in their own time or in class time if schools wish to offer it. Skills learnt through the SPARX programme include emotion identification, emotion regulation, behavioural activation (being active), recognising and challenging unhelpful thoughts and practical problem-solving. This intervention is delivered in a game format, where participants begin by choosing a personalised avatar. The gaming component sees the participant undertaking skill-building challenges in the context of a fantasy world, where the aim is to restore balance in a world taken over by gloomy, negative, automatic thoughts. The gaming component is supplemented by direct instruction, education and activities (or homework) provided by a ‘guide’ avatar, who relates the content of the programme to users’ real-life experiences.

#### Sleep Ninja

Sleep Ninja is a smartphone app based on CBT-I, which is the gold standard, evidence-based treatment for insomnia. Sleep Ninja was developed in collaboration with young people, and has been tested for feasibility, acceptability and preliminary effects on insomnia and depression [32, 39]. The intervention involves psychoeducation, stimulus control, sleep-focused cognitive therapy, basic sleep hygiene and behavioural activation.

Sleep Ninja takes the form of a chat-bot. When the app is open, conversation messages appear on the phone screen from the Sleep Ninja character (who acts as a sleep coach). Users interact with the Sleep Ninja by selecting from pre-determined responses, to which the Sleep Ninja is programmed to respond. Users progress through six levels or ‘belts’, starting at a white belt and working their way through to black belt status by the end of the programme. To level up to the next belt, users must complete within a 1-week period: one training session (which takes approximately 5–10 min); and 3 nights of sleep tracking using a sleep diary within the app. At the completion of each level, users are provided with a brief report card and graph summarising their self-reported sleep over that period. There are additional optional app features that users can access, which include a meditation recording, extra sleep information, quick sleep tips and an easy way to send app information to parents. If participants agree, the app sends a reminder each morning to enter sleep tracking from the night before, a reminder an hour before bed to begin winding down in preparation for sleep and a final reminder at bedtime for users to go to bed. Participants will have 6 weeks to complete the Sleep Ninja programme in their own time on their personal smartphones.

#### Strategies to enhance and monitor adherence

To enhance adherence, digital notifications and reminders will be sent to participants reminding them to activate, visit and use the app/s as intended. Schools will provide class time to complete some of the activities contained in the apps (minimum of four class sessions), with the rest set for homework. School staff will provide verbal reminders and encouragement to students to use the Future Proofing App and, for intervention arm schools, the first intervention app (Stage I; SPARX) during the intervention implementation period. There will also be a $20 one-off incentive to cover any connectivity and data costs, delivered post intervention, to participants in both arms, which may serve to improve adherence. Adherence to the intervention app/s will be monitored by evaluating usage data that are collected automatically by the apps while they are active. Usage data are available at the individual level and consist of when and how many times users enter the apps, how long they spend in the app, how many modules they complete and, for Sleep Ninja only, any sleep tracking data that are input by the user.

### Outcomes

The administration schedule for each of the assessment measures described in the following is presented in Table 1.

### Primary outcome measure

#### Patient Health Questionnaire—Adolescent Version

The primary outcome measure is depression severity measured at 12 months post baseline using the Patient Health Questionnaire for Adolescents (PHQ-A) [38]. PHQ-A depression scores will also be collected at baseline, 6 weeks post intervention (Stages I and II) and at 6, 24, 36, 48 and 60 months post baseline. The PHQ-A is a validated modification of the PHQ-9 for adolescents, a nine-item self-administered depression severity screening and diagnostic tool based on DSM-IV criteria. The scale assesses the frequency of occurrence of nine depression symptom criteria during the previous 2 weeks, with items rated on a 4-point scale ranging from 0 (‘Not at all’) to 3 (‘Nearly every day’). Total scale scores on the PHQ-A depression scale can range from 0 to 27, with higher scores reflecting more severe depressive symptoms. The accepted clinical cut-off points are as follows: a score of 0–4 indicates nil to minimal symptoms, 5–9 indicates mild symptoms, 10–14 indicates moderate symptoms, 15–19 indicates moderately severe symptoms and 20–27 indicates severe symptoms. The psychometric quality of the PHQ-9 and PHQ-A is well established [38, 40].

### Secondary outcome measures

Secondary outcomes will be monitored for the duration of the trial at baseline, 6 weeks post intervention (Stages I and II) and the 12-month, 24-month, 36-month, 48-month and 60-month follow-up assessments (see Table 1 for the specific measurement occasions corresponding to each outcome measure). The presence of mental health symptoms will be determined by established cut-off points relevant to each scale. Analyses will be comparisons of mean scores between the intervention and control arms at each time point. The following measures will be used to assess the secondary outcomes.

#### Distress Questionnaire-5

The Distress Questionnaire-5 (DQ-5) [41] is a five-item brief screening tool for identifying general psychological distress. Respondents rate each item on a 5-point scale, ranging from 1 (‘Never’) to 5 (‘Always’). The total scores on the scale range from 5 to 25, with higher scores indicating greater psychological distress. The scale has demonstrated optimal to high internal consistency and convergent validity, and has been found to be more accurate in identifying psychological distress than some other commonly used screeners [41, 42].

#### Spence Children’s Anxiety Scale Short-Form (including Generalized Anxiety and Social Phobia subscales)

The Spence Children’s Anxiety Scale Short-Form (SCAS-SF) is an eight-item brief measure of anxiety for children and adolescents, based on the SCAS [43]. The SCAS was designed to measure the severity of children and adolescents’ anxiety symptoms based broadly on DSM-IV criteria for anxiety disorders [44]. Respondents rate the degree to which they experience each symptom on a 4-point frequency scale, ranging from 0 (‘Never’) to 3 (‘Always’). Total scale scores on the eight-item SCAS-SF can range from 0 to 24, with higher scores reflecting greater anxiety. The SCAS Social Phobia (six items; total score = 0–18) and Generalised Anxiety (six items; total score = 0–18) subscales will also be administered in this trial to provide specific validated measures of these common anxiety disorders in adolescents. The SCAS has demonstrated high internal consistency and satisfactory test–retest reliability [43]. The SCAS has also been reported to show both convergent and divergent validity [45].

#### Insomnia Severity Index

The Insomnia Severity Index (ISI) is a psychometrically sound, seven-item self-report measure of insomnia symptoms over the previous 2 weeks [46]. Responses are reported on a Likert scale ranging from 0 (‘Not at all’) to 4 (‘Very’), producing total scores of 0–28. Cut-off scores are as follows: 0–7 reflects no clinically significant insomnia, 8–14 indicates subthreshold insomnia, 15–21 suggests moderate severity insomnia and 22–28 indicates severe insomnia. The ISI was designed for use in adults but has been widely administered to, and validated in, adolescent samples [47, 48].

### Additional outcome measures, potential mediators and risk factors

The following additional outcome measures will be examined and reported separately from the primary and secondary outcomes. Also included are variables which may mediate outcomes in the intervention arms. Potential risk factors for the development and maintenance of mental health disorders are also included (see Table 1 for the specific measurement occasions corresponding to each measure).

#### Suicidal Ideation Attributes Scale

The Suicidal Ideation Attributes Scale (SIDAS) is a recently developed brief measure of suicidal ideation severity in the past month. Using a general population sample, it has demonstrated high internal consistency and good convergent validity [49]. The SIDAS consists of five questions pertaining to frequency of thoughts, controllability of thoughts, closeness to attempt, level of distress associated with the thoughts and impact on daily functioning. Each item is assessed on a 10-point scale. Endorsement of item 3 (‘How close the individual has come to making an attempt in the last month’) will trigger the trial’s risk management procedure (see details later). A total scale score is calculated by summing item scores and can range from 0 to 50. Higher scores indicate higher levels of suicidal ideation severity.

#### Youth Risk Behaviour Survey

The Youth Risk Behaviour Survey (YRBS) was designed to assess health risk behaviours among secondary school students. Three items from the YRBS will be used in the current trial to assess suicide-related behaviours (thoughts, plans and attempts) in the past 12 months, for which participants indicate a ‘Yes’ or ‘No’ response. Studies have shown that the suicidality items demonstrate both substantial reliability [50] and good convergent and divergent validity in a secondary school sample [51].

#### Strengths and Difficulties Questionnaire

The Strengths and Difficulties Questionnaire (SDQ) [52] is a widely used behavioural screening questionnaire for children and adolescents 4–17 years old. The SDQ consists of 25 items divided between five subscales: Emotional Symptoms, Conduct Problems, Hyperactivity/Inattention, Peer Relationship Problems and Prosocial Behaviour. Respondents indicate on a 3-point Likert scale the extent to which each item applies to them, using the options 0 (‘Not true’), 1 (‘Somewhat true’) or 2 (‘Certainly true’). Total scale scores on each of the subscales can range from 0 to 10. A higher score is indicative of more problems for all subscales, except for the prosocial scale, where higher scores correspond to fewer difficulties in prosocial behaviour. The SDQ has demonstrated good internal consistency across studies [53, 54].

#### Screen for Disordered Eating

The Screen for Disordered Eating (SDE) [55] was recently developed to screen for eating disorders. The SDE comprises five items, to which respondents indicate whether they experience any disordered eating on a dichotomous scale (‘Yes’ or ‘No’). An individual is screened as positive if he/she endorses two or more items. In the primary care setting, this measure has demonstrated good discriminative accuracy [55].

#### Self-Harm Questionnaire

The Self-Harm Questionnaire (SHQ) [56] was designed to improve identification of self-harm in adolescents. The complete questionnaire consists of three screening questions enquiring about any past incidents of self-harming behaviour or thinking, followed by 12 additional questions that are only presented to adolescents reporting previous self-harm. Among a sample of psychiatric service inpatients and outpatients, the SHQ has demonstrated good concurrent and predictive validity [56]. To assess self-harm prevalence and frequency in the current trial, only screening item 3 will be administered in order to assess past episodes of self-harm (‘Have you ever actually harmed yourself on purpose? For example, have you ever cut yourself or taken an overdose and it was not an accident?’). Participants respond to this item on a 4-point scale of ‘No’, ‘Yes, once’, ‘Yes, two, three or four times’ and ‘Yes, five or more times’. This item will allow the assessment of the prevalence of self-harm and its frequency.

#### Alcohol Use and Substance Use questionnaires

The Alcohol Use questionnaire included in this trial was originally adapted from the School Health and Alcohol Harm Reduction Project [57] and used in the Climate Schools Projects [58], which are Australian school-based trials aimed at reducing alcohol and cannabis use. The questionnaire includes a standard drink diagram and nine items assessing age of first use, alongside frequency and quantity of alcohol use. An additional questionnaire assessing Other Substance Use was adapted from the Australian Institute of Health and Welfare 2007 National Drug Strategy Household Survey. The questionnaire contains five items assessing recency of substance use, with a specific focus on cannabis (additional items on age of first use and frequency of use), and also assesses tobacco, amphetamine, ecstasy, hallucinogens, sedatives and inhalant use.

#### Adolescent Psychotic-Like Symptom Screener

The Adolescent Psychotic-Like Symptom Screener (APSS) [59] is a seven-item measure designed to identify people who are at increased risk of future clinical psychotic disorder. In this study, only three items will be administered to assess paranoia, auditory and visual hallucinations. For each question, there are three possible responses: ‘Yes, definitely’, ‘Maybe’ and ‘No, never’. Responses are scored 1, 0.5 and 0 respectively. Higher total scale scores on this measure are indicative of greater psychotic-like symptoms. In the general population, this instrument has demonstrated good sensitivity and specificity in identifying young adolescents with psychotic-like experiences [59].

#### Pittsburgh Sleep Quality Index

The Pittsburgh Sleep Quality Index (PSQI) [60] was designed to assess sleep quality and disturbances over a 1-month interval. The PSQI consists of 19 items, which are used to compute seven component scores: sleep quality, sleep latency, sleep duration, habitual sleep efficiency, sleep disturbances, use of sleep medications and daytime dysfunction. Each item is weighted equally on a scale that ranges from 0 (no difficulty) to 3 (severe difficulty) scale. The seven component scores are then summed to yield a global score, ranging from 0 to 21; higher scores indicate worse sleep quality. The PSQI has demonstrated acceptable to good internal homogeneity, test–retest reliability and convergent validity across studies [60–62].

#### Child Health Utility 9D

The Child Health Utility 9D (CHU-9D) [63] is a nine-dimension generic preference-based measure designed to assess child and adolescent health-related quality of life and suitable for application in economic evaluation. Current child/adolescent health-related quality of life is assessed across the domains of worry, sadness, pain, tiredness, annoyance, school, sleep, daily routine and activities. Each dimension is rated on a 5-point response scale ranging from ‘no’ to ‘severe impairment’. Responses are then converted to utilities on the 0–1 dead to full health quality-adjusted life years (QALY) scale using a preference-weighted scoring algorithm [64]. Previous validation studies with adolescents from the community and mental health services have demonstrated that the self-complete instrument has acceptable internal consistency and convergent validity for children and adolescents aged 7–17 years [65–67].

#### Short Warwick–Edinburgh Mental Well-Being Scale

The Short Warwick–Edinburgh Mental Well-Being Scale (SWEMWBS) [68] is a shortened seven-item version of the 14-item Warwick–Edinburgh Mental Well-Being Scale (WEMWBS), which was developed to assess mental well-being in the general population. The SWEMWBS consists of seven statements about thoughts and feelings over the past 2 weeks. Ratings are made on a 5-point Likert scale (1 = ‘None of the time’, 2 = ‘Rarely’, 3 = ‘Some of the time’, 4 = ‘Often’, 5 = ‘All of the time’). Total scale scores are calculated by summing item scores and transforming the total score using a conversion table. Total scores can range from 7 to 35. A higher score indicates a higher level of mental well-being. The SWEMWBS has demonstrated adequate reliability and validity across studies [69, 70].

#### Demographic information

At the baseline assessment, participants will be asked to provide their date of birth, postcode, country of birth, language spoken at home, who they live with at home, Aboriginal and Torres Strait Islander status, and socio-economic status [71].

#### Height and weight

Self-reported height (cm) and weight (kg) will be provided.

#### Big Five Personality Inventory

The Big Five Personality Inventory (BFI-10) [72] is a 10-item scale measuring the Big Five personality traits: Extraversion, Agreeableness, Conscientiousness, Neuroticism and Openness. The scale was developed based on the 44-item Big Five Inventory (BFI-44) [73]. Respondents indicate their level of agreement with each described trait on a 5-point scale, ranging from 1 = ‘Disagree strongly’ to 5 = ‘Agree strongly’. Total subscale scores are calculated by summing item scores (two items per subscale). Subscale scores can range from 2 to 10. The BFI-10 has demonstrated acceptable test–retest reliability and good construct validity across studies [72, 74].

#### School connectedness

School connectedness will be assessed using questionnaire items developed by the Organisation for Economic Co-operation and Development (OECD) Programme for International Student Assessment [75]. Six items will be administered, which are rated on a 4-point scale from 1 (‘Strongly agree’) through to 4 (‘Strongly disagree’). Total scale scores can range from 6 to 24, with higher scores reflecting greater school connectedness.

#### Schuster Social Support Scale

The Schuster Social Support Scale (SSSS) [76] is a 15-item measure of positive and negative interactions with family, friends and spouse. In the current study, 10 items will be administered to assess interactions with family and friends only. Each item is rated on a 4-point scale ranging from 0 (‘Never’) through to 3 (‘Often’). Scores are interpreted per category, for friends and family, with higher scores on the supportive interactions scales indicative of more supportive interactions, and higher scores on the negative interactions scales indicate more negative interactions.

#### Maladaptive Facebook Usage Scale (adapted)

The Maladaptive Facebook Usage Scale [77] is a seven-item measure of maladaptive Facebook usage, which assesses an individual’s tendency to undertake negative social evaluations and social comparisons when they use Facebook. This scale has demonstrated good test–retest reliability and convergent validity [77]. In this trial, the scale has been adapted to incorporate social media more broadly. A screening item has been added asking respondents to nominate which social media platforms they use at least once per week. Options include: Facebook, Instagram, Snapchat, Tumblr, Twitter, YouTube, Reddit, Other and ‘I don’t use social media’. The seven items of the Maladaptive Facebook Usage Scale were adapted to apply to any social media platform (e.g. ‘I tend to read the social media status updates of others to see if they are feeling the way I am’ and ‘Reading the social media status updates of others tends to make me feel down on myself’). Items are rated on a response scale from 1 (‘Strongly disagree’) to 7 (‘Strongly agree’). Total scale scores can range from 7 to 49. Higher scores indicate greater tendencies to seek online social comparisons and negative evaluations.

#### Gender identification and sexual identification/preferences

Gender identification (two items: sex at birth and current gender identity) and sexual identification and preferences (one item for each) will be examined at baseline and annually between 12-month and 60-month follow-up.

#### Romantic relationships

Two items will be presented at multiple time points to assess romantic relationships. These items will ascertain the number of special or important romantic relationships in the past year, as well as the number of break-ups in the past 12 months.

#### Sexual behaviour

A series of questions will be presented at the 24-month follow-up when participants are in Year 10 (aged 15–16 years) to assess sexual behaviour, taken from the National Survey of Australian Secondary Students and Sexual Health [78]. These items will assess experience and age of first ‘making out’, intimate genital touching and sex, as well as number of people they have had sex with during the past year and frequency of condom use. This section will include an initial gating item assessing history of intimate sexual contact. Those who do not endorse this item will not receive the other sexual behaviour items.

#### Trauma Behavioural Risk Factor Surveillance System—Adverse Childhood Experience Module

The Trauma Behavioural Risk Factor Surveillance System—Adverse Childhood Experience Module (BRFSS-ACE) [79] consists of 11 items that assess exposure to nine types of adverse childhood experiences in the first 18 years of life, including: verbal abuse, physical abuse, sexual abuse, household mental illness, household alcohol abuse, household drug abuse, domestic violence, parental separation/divorce and incarcerated family members. The responses are dichotomised to indicate exposure and summed to create an ACE score ranging from 1 to 8 for each subdomain, with higher scores indicating greater exposure. Previous studies have demonstrated that this instrument has adequate internal consistency and validity [80, 81]. In this trial, the complete BRFSS-ACE will be administered at 48-month follow-up when participants are near 18 years of age. A modified version of the questionnaire consisting of eight items will be administered at baseline when participants are approximately 13 years old. This modified scale excludes items on physical and sexual abuse, and includes additional items assessing out-of-home or foster care and feelings of endangerment or physical harm.

#### History of mental health diagnosis

One item will assess the lifetime history of diagnosed mental health problems (major depression, social anxiety disorder, generalised anxiety disorder, obsessive compulsive disorder, panic disorder, alcohol use disorder, substance use disorder, attention deficit hyperactivity disorder, post-traumatic stress disorder, schizophrenia/psychosis).

#### Hospitalisation

One item will assess hospitalisation in the previous 12 months and will ask participants to distinguish between hospitalisation for physical and mental health problems.

#### History of disability diagnosis

One item will assess the lifetime history of diagnosed disability (autism or Asperger’s syndrome, intellectual disability, specific learning disability, Tourette syndrome, cerebral palsy, acquired brain injury, other neurological disability, hearing impairment, visual impairment).

#### Pubertal development

A separate set of questions for females and males will be used to assess pubertal development (e.g., ‘at what age did you get your first period/did your voice begin to break?’). Participants will also be provided with line drawings of stages of pubertal development (Tanner Stages) and asked to rate their current stage of physical development against these images.

#### Bullying

Bullying items were drawn from previous school-based trials. The three items examine whether participants have been bullied, have been cyber-bullied or have bullied others in the past 12 months. Items are responded to on a 5-point frequency scale from 1 (‘Not at all’) to 5 (‘Most days’).

#### App use and feedback surveys

App use and feedback surveys for both the Future Proofing app and the SPARX app will be administered at the post-assessment time point. The Future Proofing App Survey was developed for this study and has nine items which assess app task preferences and reasons for discontinued use. The SPARX Feedback Survey is an 11-item survey that has been used previously to assess SPARX use [23] and app acceptability, and asks participants to select any skills they learnt from using the app.

### Smartphone-collected data

Additional data will be collected directly via smartphones through a study app developed for this study (the Future Proofing App), as these data cannot be collected from the self-report questionnaires outlined earlier. Three forms of data will be collected, which include actively collected data, passively collected data and app usage data. Actively collected data will assess self-reported mood ratings, voice samples and cognitive tasks. This form of data will overcome biases in retrospective reporting of mood and allow for an investigation of whether changes in voice are related to changes in mental health states. Cognitive tasks include two measures of executive function presented in affective and neutral contexts. Specifically, a measure of affective shifting [82] and the Affective Digit Span [83] and a typing speed task, which will allow for a mechanistic assessment of the way cognitive function affects psychological outcomes in adolescents.

Passively collected smartphone data will involve the collection of location data (GPS) and movement data (accelerometry), which will be used to investigate whether mental health changes can be predicted from location and activity data. Smartphone sensor data will be collected during the first 3 months of the study, and at each annual assessment point for 3-month periods. App usage data will allow for assessment of app use and completion, and time spent using the study app/s.

### Linked data

Data relating to participants’ academic outcomes, physical health, utilisation of health services, infant development, births and deaths will be linked from extant Australian government administrative datasets by an authorised agency of the New South Wales Ministry of Health, the Centre for Health Record Linkage (CheReL, www.cherel.org.au). Informed consent from each participant will be obtained prior to linkage and databases will be linked using personal identifiers such as names, dates of births, addresses and hospital identification numbers with probabilistic methods. Linked data will be provided to the researchers in a de-identified manner and will be used to determine related health and other outcomes for the participant, including birth and perinatal data, educational outcomes in standardised curriculum-based tests, and hospitalisation and mortality outcomes. Linkage will occur within the first 12–24 months of the study (at the end of 2020) and will be updated periodically every 2 years until the trial concludes.

### Procedure and participant timeline

Figure 1 shows the participant timeline and flow. An initial preparatory phase and first school visit will solicit parental and student consent, ensure that electronic study questionnaires are accessible by consented students and ensure that the appropriate study apps are installed on each student’s device. Each subsequent school visit will coincide with an assessment occasion. During these visits, students will access study questionnaires via a secure online portal, accessible using their mobile number and a one-time password sent via SMS. Study personnel will attend these school visits to assist with technical issues and participants’ questions. Following completion of baseline questionnaires, participants in the intervention condition will be instructed to commence the intervention both during class time and in their own time, and will receive notifications and reminders over the following 6 weeks to do so. Schools are required to schedule four 20-min in-class sessions for intervention completion but may choose to hold additional sessions (up to seven). Participants in the control condition will also be provided with equivalent class time to complete activities on the Future Proofing app. Students who do not complete assessments in school will also have the option to do this in their own time. Students will have up to 4 weeks following the scheduled assessment date to complete questionnaires at each time point, after which the assessment surveys will be locked and no longer accessible. During this 4-week window, automated electronic reminders will be sent to students to prompt completion, if required.

### Sample size

Separate sample size estimates were derived to determine the numbers of participants needed to meet the aims of each trial stage. Power was set at 0.80, α = 0.05 (two-tailed), and a correlation of 0.5 assumed between baseline and endpoint symptom scores. To account for possible clustering effects at Stage I (i.e., participants from the same school having prognostically relevant characteristics and outcomes more alike than between schools), a design effect [84] was calculated assuming an intraclass correlation coefficient (ICC) of 0.03 (based on previous school-based studies) [85] and a cluster size of 50 students, yielding a design effect of 2.47. In order to detect a mean standardized difference of 0.3 between conditions at Stage I, a sample of 870 participants would be needed (435 per arm). Allowing for up to 30% attrition, recruitment of 1244 participants would be required (622 per arm).

Using similar parameters for Stage II (without a design effect) would require recruitment of 368 participants in total to detect the same effect size. However, participants in this stage must meet eligibility requirements regarding elevated symptoms and failure to respond to the first-stage intervention. They must also be willing to participate. Accordingly, a scenario involving recruitment of 10,000 participants at Stage I was considered, assuming 7000 participants would have endpoint data available at 12 months (allowing for 30% attrition). Conservatively estimating 20% having PHQ-A scores ≥ 10, 25% showing a reduction due to the Stage I intervention and that 50% of those eligible to enrol at Stage II do so, 525 participants would enter this stage II, with endpoint data available for at least 368 (allowing for up to 30% attrition). This sample would enable the detection of differences in changes in symptomatology scores of comparable size to those in Stage I of the trial (*d* = 0.29).

Given that a target sample of 10,000 is substantial, if the numbers of participants are not sufficient to reach the Stage II target, then supplementing this sample with additional young people specifically targeted based on PHQ-A scores that fall in the top 20% of population scores to ensure that this stage of the trial has comparable power to Stage I to detect an intervention effect will be considered. This will ensure that sufficient numbers are included at both stages of the trial. Should the target be met, Stage I of the trial will have much higher power to detect changes in symptom scores and would be adequately powered to detect differences in incidence based on PHQ-A diagnosis criteria.

Exploratory analyses for moderating and mediating effects will be feasible at both stages. Stage II will have power to detect small to medium mediation effects [86]. Power to detect medium-size effects (0.5 SD) in moderation analyses will be maintained for subgroups down to approximately 120 participants. Power will be enhanced in longitudinal modelling (growth curves) which use multiple occasions of measurement to estimate rates of change [86].

### Randomisation

For Stage I, cluster randomisation (at the school level) will be employed for administrative convenience, to avoid control condition contamination, and for the ecological validity of providing the intervention at the cluster level. Schools will be randomised after they are recruited into the trial. The trial statistician who is not involved in the day-to-day running of the trial will perform the randomisation, and the identity of the school will be concealed from the statistician.

Schools will be randomised with a 1:1 allocation, as per a computer-generated randomisation schedule. Balance between the trial arms will be achieved by stratifying based on school size, school location (metropolitan vs. regional), school type (co-educational or gender selective) and Index of Community Socio-Educational Advantage (ICSEA) level. For Stage II, individual-level randomisation with a 1:1 allocation using a computer-generated randomisation schedule stratified by gender and depression severity scores will be employed. This procedure is automated through the Black Dog Institute research platform. Permuted block randomisation will be used at both stages but will not be disclosed to ensure concealment. Allocation to arms is not directly communicated to schools. However, because the study has no control intervention, schools, participants and study operational staff will be aware of school allocation to the intervention arm. With the exception of the trial data manager, other individuals not involved in the day-to-day running of the trial will remain blind to allocation. All outcome assessments are conducted electronically and not subject to assessor bias. Unblinding at the conclusion of analysis will be performed by the trial data manager with authorisation from the Trial Steering Committee.

### Participant risk management protocol

An independent Data and Safety Monitoring Committee (DSMC) has been established to monitor the quality of trial data and the safety of research participants. The DSMC will be responsible for safeguarding the interests of participants through regular monitoring, including participant safety and adverse events. This group will be responsible for monitoring the efficacy of the interventions being tested on primary outcomes, as well as the overall conduct of the study, including recruitment, protocol compliance, accuracy and completeness of data collection. This group will also provide recommendations with respect to continuing, modifying or terminating the trial, on the basis of feasibility or safety concerns, and will have access to unblinded data. This group can recommend the trial team be unblinded if there are serious safety concerns and meets every 6 months to review trial conduct. There is no anticipated harm from this trial, as it involves a population-based sample and evidence-based interventions. However, should there be any unanticipated harms, this will be monitored by the DSMC, who will provide recommendations about care for affected participants.

All students involved in the trial will be assessed for suicidal thinking and behaviour at each assessment point, using the SIDAS and the YRBS. If participants indicate serious suicidal thinking, plans or behaviour on study surveys, an alert will be triggered whereby the research team and school counsellor are notified immediately using a purpose-built study portal. School counsellors will then follow-up with students within 48 h to offer immediate support, or refer on when necessary, which is recorded in the portal. The research team will monitor this portal and directly contact any counsellors who have not indicated follow-up with students. If students have changed or left schools, parents will be notified. At the 48-month assessment point, when students are in their final year of school, they will also be asked about history of sexual abuse. If students indicate an experience of sexual abuse, this same process of notifying the school counsellor will be followed, and schools will assume the duty of care for mandatory reporting requirements. This information will be communicated to relevant university and school ethics committees, as well as the DSMC following each assessment point.

### Data collection and management

All research data collected in this trial will be stored using a unique participant ID code. A list of identifiable participant information associated with each ID code will be stored separately from the research data. The privacy, security and ownership of the research data will be maintained, and re-identifiable data will not be stored or accessible by another organisation. Access, storage and transmission logs will be recorded and regularly reviewed for anomalies. Annual audits will be conducted by the trial data manager to ensure compliance with data security processes outlined by UNSW and Black Dog Institute.

The data that will be collected include questionnaire data, mobile phone data and linked data. Coded questionnaire outcome data will be stored securely on the Black Dog Institute online research platform until ready for export. The research platform is stored on UNSW servers and supported by enterprise access controls and 256-bit encryption or higher. Data will be exported from the research platform into Microsoft Excel following assessments so that they can be checked by the data manager for data quality and accuracy. After checking, data will be exported into appropriate statistical software for analysis. Data collected by mobile phone apps will be encrypted and transmitted to a secure database hosted by Google Cloud Services in Australia. Data will be securely removed and transferred on a scheduled basis to UNSW servers by the trial data manager. Access to Google Cloud Services and the UNSW server will require authentication and will be restricted to the data manager and named members of the research team. The data manager will be responsible for extracting and securely transferring data to the research team. Only researchers whose analyses require access to the specific dataset collected from each questionnaire, app and linkage data source will be able to access those data. Linked datasets will be subject to the requirements set out by linking agencies for storage on UNSW servers using the already outlined procedures.

### Analysis

#### Primary outcome

Analysis of the primary outcome will be undertaken using an intention-to-treat approach including all participants randomised regardless of intervention received, controlling for baseline differences when appropriate. The primary analysis will be conducted using planned contrasts comparing a change in depression scores on the PHQ-A from baseline to 12 months between the trial arms (SPARX intervention vs. control), using a mixed-effects model repeated-measure analysis (MMRM). MMRM is preferred due to the ability of this approach to include participants with missing data without using discredited techniques such as last observation carried forward [87, 88]. School will be included as a random effect to evaluate and accommodate clustering effects. Variables used in determining allocation balance will be evaluated and retained in analyses where they are significant or quasi-significant. An unconstrained variance–covariance matrix will model within-individual dependencies. Transformation of scores, including categorisation, may be undertaken to meet distributional assumptions and accommodate outliers.

#### Secondary and additional outcomes

Secondary and additional outcome analyses will involve contrasts comparing change on secondary (DQ-5, SCAS, ISI), and other (e.g., SIDAS) outcomes from baseline to other occasions of measurement, using an MMRM approach, as already described.

#### Additional analyses

Subsidiary complier analyses will be undertaken to compare individuals who complete the intervention relative to those who do not, in both trial stages. Regression models will be used to examine risk factors for symptoms of psychopathology across the study measurement points in the control arm. Mediation analyses will be explored using structural equation modelling. Machine learning approaches will be used to link multiple data types with outcomes to discover novel factors which predict outcomes in terms of specific disorders and their progression as well as investigate individual patterns of data that predict individual mental state or symptom trajectory. Cost-effectiveness analyses will be conducted at the primary endpoint and the final measurement point. Data will be linked to existing records, and education and health outcomes will be reported.

#### Interim analyses

The percentage of participants meeting PHQ-A depression caseness, scores and reporting of suicidality at each assessment within both groups will be measured and reviewed by the DSMC in order to monitor relative levels of deterioration (accounting for baseline levels), for safety purposes.

### Ethics and dissemination

This study has ethical approval from the University of New South Wales Human Research Ethics Committee (HC180836) and NSW Government State Education Research Applications Process Approval (SERAP 2019201), and has applied for approval from Catholic dioceses and the Tasmanian Department of Education. The trial is subject to annual progress reviews with these ethics bodies. The trial is registered with the Australian New Zealand Clinical Trials Registry (ANZCTRN12619000855123). All protocol amendments will be subject to approval by relevant ethics committees and listed on the ANZCTR registry. All trial findings will be presented in aggregate format so that no individual-level data will be presented.

Public access to the full protocol can be granted from the authors on reasonable request. Access to participant-level dataset will be subject to governance processes set up around a data repository which will contain data from this study. This will be open for researchers to apply for access.

Trial findings will be communicated using lay language and will be made available to participating schools for publication in school newsletters and/or school websites. Participants and parents will also be provided with these findings via email. Regular trial progress updates will be provided by the research team to schools for distribution through the school community, at their discretion. These findings will also be provided to other stakeholders in the wider community, including to the government in policy documents, school counsellor bodies, teacher groups and mental health groups. All findings will be provided at aggregate level. The results of the trial will be disseminated via peer-reviewed publications in scientific journals and conferences. No restrictions have been imposed on the dissemination of information by funders.

## Discussion

The prevention of depression is critical if the burden of disease is to be reduced. This study will be the first to examine the prevention of depression in young people at scale, using easy-to-access, convenient and private mobile-phone applications. Efficacy of depression prevention has been established [17, 19]. What The Future Proofing Study provides is a large definitive trial investigating the impact of delivering evidence-based prevention programmes like CBT at scale, in real-world settings. A large-scale trial such as this can provide insight into scaling and implementation processes, which can form the basis of an ongoing delivery and dissemination framework. The current study meets this need by examining a depression-prevention programme, delivered by mobile phone applications, with access facilitated in the school environment that is where young people spend most of their time. This means that, if successful, schools could become the setting in which access to these programmes is rolled out continually, future proofing and inoculating all young people who go through the school system against mental illness.

There are several other features of this trial that make it novel. The use of a two-staged design, such that those who show symptoms of depression at the primary endpoint are randomised to receive a second intervention targeting insomnia, is innovative. This second intervention offers an alternative way to reduce risk for depression onset. The use of smartphone apps as the primary intervention delivery mechanism is new in this area [27, 89] and promises reduced costs and access barriers compared to traditional face-to-face and health professional-led programmes. The inclusion of smartphone-collected objective data adds another dimension to the trial, allowing for machine learning approaches to explore whether activity and location data are associated with, or predictive of, changes in depression and other mental health symptoms. Moreover, linking this rich dataset to government education and health records will permit direct inferences to be made about the impact of mental illness on health and academic outcomes. Finally, the continuation of follow-up assessments for 5 years means that the long-term effects of the interventions on depression, anxiety, distress, insomnia and additional outcomes can be assessed. The results of The Future Proofing Study stand to provide a major contribution to the field of digital mental health prevention programmes, and knowledge about the population-level impact of preventing depression using these methods.

## Supplementary information

**Additional file 1.** SPIRIT 2013 Checklist: Recommended items to address in a clinical trial protocol and related documents.

## Data Availability

Not applicable.

## Abbreviations

CBT: Cognitive behaviour therapy
CBT-I: Cognitive behaviour therapy for insomnia
RCT: Randomised controlled trial
